# Long‐Term Survival With Minimum Side‐Effects in a Post‐Op Case of Carcinoma Esophagus With Multiple Recurrences in Mediastinum Treated With Radical Re‐Radiotherapy in Mediastinum—A Case Report

**DOI:** 10.1002/ccr3.71520

**Published:** 2026-02-06

**Authors:** Ajay Kumar Choubey, Pritam Mondal, Shubham Dokania, Sambit Swarup Nanda, Shreya Jain, Minesh Patel, Ashutosh Mukherji, Satyajit Pradhan

**Affiliations:** ^1^ Department of Radiation Oncology Mahamana Pandit Madan Mohan Malaviya Cancer Centre Varanasi India; ^2^ Homi Bhabha National Institute Navi Mumbai India

**Keywords:** case report, esophagus, recurrence, re‐radiotherapy

## Abstract

Loco‐regional recurrence after radical surgery in esophageal cancer is common. However, there is no universally accepted guideline for managing such loco‐regional recurrences. Even after multiple local recurrences in the mediastinum, a patient can consolidate safely by re‐radiotherapy in the mediastinal region with minimal late morbidity by judicious use of radiotherapy, if there is an acceptable time gap between two radiotherapy periods.

## Graphical abstract

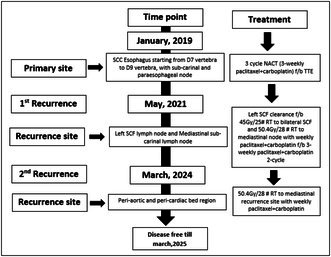

**Abbreviations**
AJCC: American Joint Committee on Cancer
CECT: contrast enhanced computed tomography
CRM: circumferential resection margin
CTCAE: common terminology criteria for adverse events
CTV: clinical target volume
EBUS‐TBNA: endobronchial ultrasound‐guided transbronchial needle aspiration
EQ D2: equivalent dose at 2 Gy per fraction
FDG: 2‐[18F] fluoro‐2‐deoxy‐D‐glucose
FNAC: fine‐needle aspiration cytology
GTV: gross tumor volume
Gy: Gray
NACT: neo‐adjuvant chemotherapy
nCRT: neo‐adjuvant chemo‐radiotherapy
OAR: organ at risk
PET‐CT: positron emission tomography‐computed tomography
PTV: planning target volume
RT: radiotherapy
SCC: squamous cell carcinoma
SCF: supra clavicular fossa
SRT: stereotactic radiotherapy
SUV: standardized uptake value
TPS: treatment planning system
TTE: trans‐thoracic esophagectomy
UGIE: upper gastro‐intestinal endoscopy

## 1 Introduction

Esophageal cancer ranks as the 11th most common cancer in terms of incidence and 7th most common in terms of mortality worldwide [1]. Approximately 90% of patients succumb to the disease due to progression or related complications [2]. For locally advanced, operable thoracic esophageal carcinoma, the standard of care includes neoadjuvant chemoradiotherapy (nCRT) [3] or neoadjuvant chemotherapy (NACT) [4, 5], followed by radical esophagectomy. In medically inoperable patients, definitive chemoradiotherapy serves as an alternative treatment option aimed at organ preservation. Despite curative‐intent treatment, locoregional or distant recurrences are frequently observed [6]. Regional lymph node recurrence occurs in up to 50% of patients following esophagectomy [7], and while it may be amenable to salvage or curative treatment, it significantly worsens overall prognosis. Cases of twice‐occurring regional recurrences, successfully managed with repeat curative‐intent chemoradiotherapy, and achieving complete response with minimal toxicity, are extremely rare. We present a unique case of a patient with locally advanced thoracic esophageal squamous cell carcinoma (SCC) who demonstrated long‐term survival and excellent tolerance to treatment, despite experiencing two regional recurrences, both managed with curative chemoradiotherapy.

## 2 Case History/Examination

A 46‐year‐old female from Uttar Pradesh, India, with no history of substance abuse or comorbidities, presented with complaints of gradually progressive dysphagia to solid foods over 4 months. She had no difficulty in swallowing liquids. Dysphagia to solid food was occasionally associated with a burning sensation over the chest during swallowing. On physical examination, the abdomen was found to be soft, nontender and no organomegaly was present. No supraclavicular node was palpable. On chest auscultation no abnormal sounds were found.

## 3 Methods (Investigations and Treatments)

An upper gastrointestinal endoscopy (UGIE) performed on January 26, 2019 revealed an ulcero‐proliferative growth in the lower third of the esophagus, beginning 30 cm from the upper central incisors. The endoscope could not be negotiated beyond the lesion. Biopsy confirmed a diagnosis of moderately differentiated squamous cell carcinoma. A positron emission tomography‐ computed tomography (PET‐CT) scan demonstrated 2‐[18F] fluoro‐2‐deoxy‐D‐glucose (FDG)‐avid thickening of the esophageal wall from the inferior edge of the D7–D9 vertebrae, with the GE junction appearing tumor‐free. Additionally, it revealed FDG‐avid nodes: an 8‐mm subcarinal and a 5‐mm left paraesophageal nodule. A right level II cervical lymph node (9 × 6 mm) was non‐FDG‐avid. Ultrasound‐guided fine‐needle aspiration cytology (FNAC) of the suspicious right cervical node confirmed it as a reactive node.

The patient underwent three cycles of neoadjuvant chemotherapy (NACT) with paclitaxel and cisplatin administered every 3 weeks between February 28 and April 13, 2019. Subsequently, she underwent an open transthoracic esophagectomy (TTE) with mediastinal lymphadenectomy on June 1, 2019. Histopathological examination revealed residual viable, poorly differentiated squamous cell carcinoma without necrosis. The tumor infiltrated the muscularis propria and extended into the adventitia, with evidence of lymphovascular and perineural invasion. Among 32 dissected lymph nodes, two (one in the aortopulmonary [AP] window and one in the lower paraesophageal region) showed metastatic involvement, both without extranodal extension. The proximal and distal resection margins were 4 cm and 5.5 cm, respectively, and the circumferential resection margin (CRM) was 0.7 cm from the nearest firm area. Final staging was ypT2N1, based on the American Joint Committee on Cancer (AJCC) 8th edition. The patient was subsequently kept on regular follow‐up and remained disease‐free until May 2021.

### 3.1 First Recurrence (May 2021)

**FIGURE 1:**
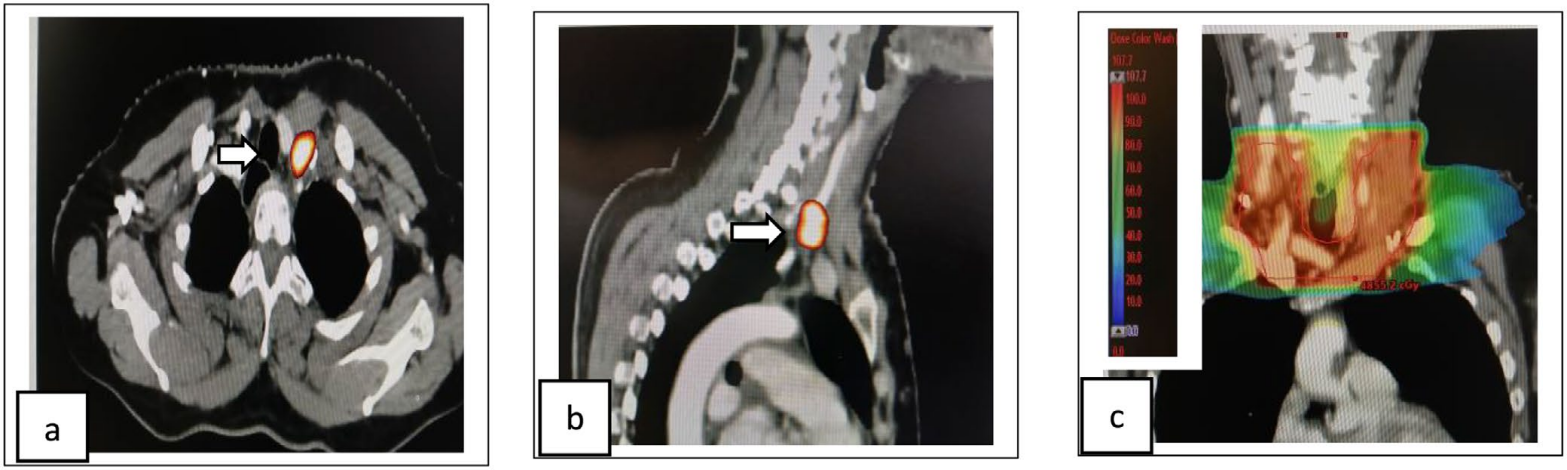
First recurrence in left SCF. (a, b) White arrow in axial and sagittal sections of CT superimposed with PET shows left SCF nodal recurrence. (c) Coronal CT section shows dose color wash of SCF region treatment volume.

**FIGURE 2:**
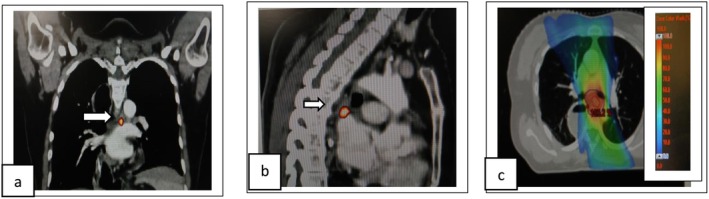
First recurrence in mediastinal nodal region. (a, b) White arrow in coronal and sagittal sections of CT superimposed with PET shows first mediastinal nodal recurrence. (c) Axial CT section shows dose color wash of mediastinal nodal treatment volume.

During routine follow‐up on May 10, 2021, the patient presented with a swelling in the left supraclavicular fossa. FNAC under ultrasound guidance confirmed metastatic squamous cell carcinoma. PET‐CT revealed an FDG‐avid left supraclavicular fossa (SCF) lymph node (2.1 × 2.8 cm), standardized uptake value (SUV_max_ 11.6) (Refer Figure 1a,b) and an FDG‐avid subcarinal mediastinal node (7 × 8 mm, SUV_max_ 6.38) (Refer Figure 2a,b). The case was reviewed in the thoracic tumor board, and because of her relatively young age and a moderate disease‐free interval, a decision was made to pursue definitive concurrent chemoradiation following SCF nodal dissection.

The patient underwent left SCF nodal clearance on June 8, 2021, with histopathology confirming metastatic squamous cell carcinoma without extranodal extension. She then received radiotherapy: 45 Gy in 25 fractions to the SCF (Refer Figure 1c) and 50.4 Gy in 28 fractions to the mediastinal nodal region (5 fractions per week over 5.5 weeks) (Refer Figure 2c). Concurrent chemotherapy included five cycles of weekly paclitaxel and carboplatin, completed on September 17, 2021, followed by two additional cycles of consolidative chemotherapy. The mediastinal dose was carefully planned to remain within safe limits for the reconstructed gastric conduit (*D*_max_ < 54 Gy). RT volume for SCF region: gross tumor volume (GTV)‐ none as SCF clearance done prior to RT, clinical target volume (CTV)‐ bilateral neck level IVa and IVb station, planning target volume (PTV)‐ CTV+ 5 mm margin (crop from body by 3 mm). RT volume for mediastinal node, GTV‐ gross node (as per FDG avidity and CT fusion), CTV‐ 10 mm margin all around (crop from anatomical boundaries), PTV‐ CTV+ 5 mm margin (Crop from body by 3 mm). The highest acute toxicities observed were Grade 2 odynophagia, Grade 1 dysphagia, and Grade 1 skin reaction (Refer Table 2). A response assessment PET‐CT 3 months post‐treatment showed a complete metabolic response. The patient resumed a regular diet with improved swallowing and was kept under close follow‐up with 3‐monthly contrast enhanced computed tomography (CECT) of thorax and abdomen and annual PET‐CT and UGIE.

### 3.2 Second Recurrence (March 2024)

**FIGURE 3:**
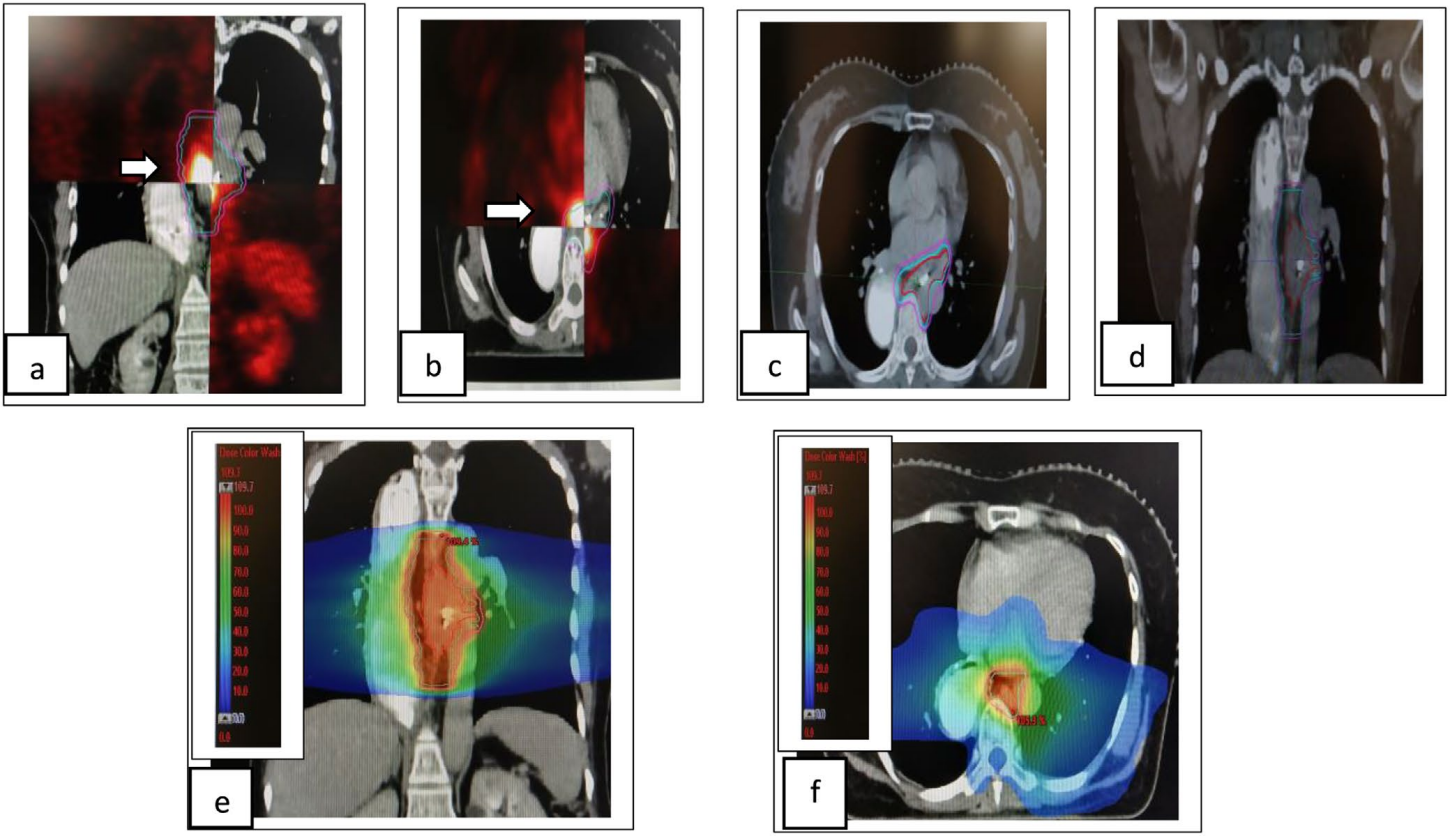
Second recurrence in the mediastinal region. (a, b) White arrow in coronal and axial sections of CT superimposed with PET shows second mediastinal nodal recurrence. (c, d) Axial and coronal CT section shows GTV (red), CTV (sky blue), and PTV (pink) for the second mediastinal recurrence. (e, f) Coronal and axial CT section shows dose color wash.

**FIGURE 4:**
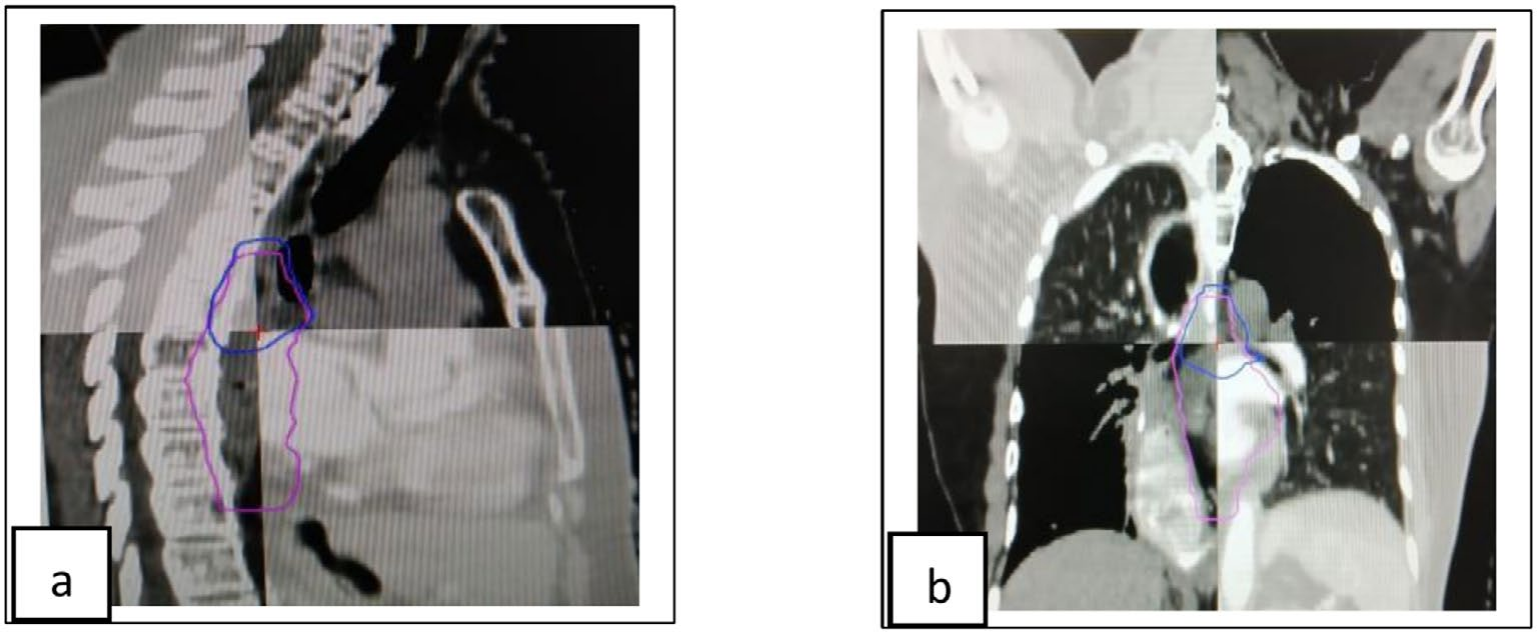
(a, b) Sagittal and coronal images show that superimpose primary RT and re‐RT planning scan; pink—PTV volume in re‐RT; blue—PTV volume of primary RT, showing overlapping volume.

On March 28, 2024, a follow‐up PET‐CT showed an FDG‐avid irregular soft tissue lesion in the posterior cardiac/peri‐aortic region (50 × 27 × 57 mm, SUV_max_ 22.53) and an FDG‐avid pre‐carinal node (SUV_max_ 4.31) (Refer Figure 3a,b). Additionally, three small lung nodules (maximum 6 mm) with nil to low FDG uptake were noted—two in the right lung and one in the left. Endobronchial ultrasound‐guided transbronchial needle aspiration (EBUS‐TBNA) of the subaortic and subcarinal nodes confirmed metastatic squamous cell carcinoma. This recurrence posed a greater therapeutic challenge due to overlap with the previously irradiated thoracic region (Refer Figure 4a,b), including the gastric pull‐up used as neo‐esophagus and adjacent organs at risk (OARs) like the heart and lungs. To minimize toxicity, the radiation field was limited to the recurrent site with an appropriate margin, avoiding re‐irradiation of the entire thoracic bed. This required precise and individualized radiation planning by the physics and clinical teams to ensure optimal tumor coverage while sparing critical structures.

**FIGURE 5:**
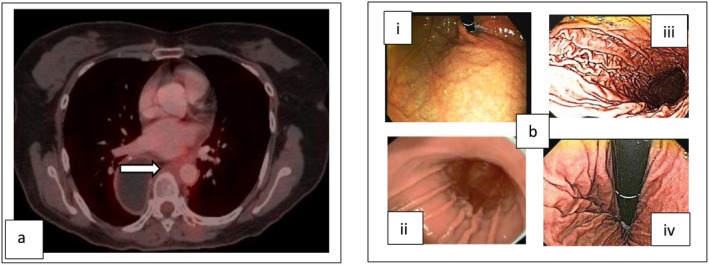
(a) White arrow in axial section of response PET‐CT after second time curative chemo‐radiotherapy shows complete metabolic response. (b) Picture shows UGIE after second time curative chemo‐radiotherapy. (i, ii) shows upper and middle gastric conduit respectively and (iii, iv) shows lower part of gastric conduit.

The patient underwent re‐irradiation of 50.4 Gy in 28 fractions over 5.5 weeks with concurrent weekly paclitaxel and carboplatin (six cycles), completed on 19th June 2024 (Refer Figure 3c–f). RT volume was, GTV‐ gross disease (As per FDG avidity and CT fusion), CTV‐ 20 mm craniocaudal and 10 mm circumferential expansion (crop from anatomical boundaries), and PTV‐ CTV+ 5 mm margin (crop from body by 3 mm). She tolerated treatment well, experiencing only Grade 1 dysphagia without odynophagia or weight loss (Refer Table 2). Follow‐up PET‐CT on September 18, 2024 showed a complete metabolic and significant morphologic response (Refer Figure 5a). The lung nodules remained stable and non‐FDG avid. UGIE showed a normal study (Refer Figure 5b).

Eclipse Treatment Planning Systems (TPS), Version 15.6 was used for planning. Treatment was executed on the TrueBeam machine 2.7 linear Accelerator (Model SDC, Varian Medical Systems) with a multileaf collimator (MLC) leaf width of 5 mm at the isocentre, using 6 MV photons.

## 4 Conclusions and Results (Outcome and Follow‐Up)

**FIGURE 6:**
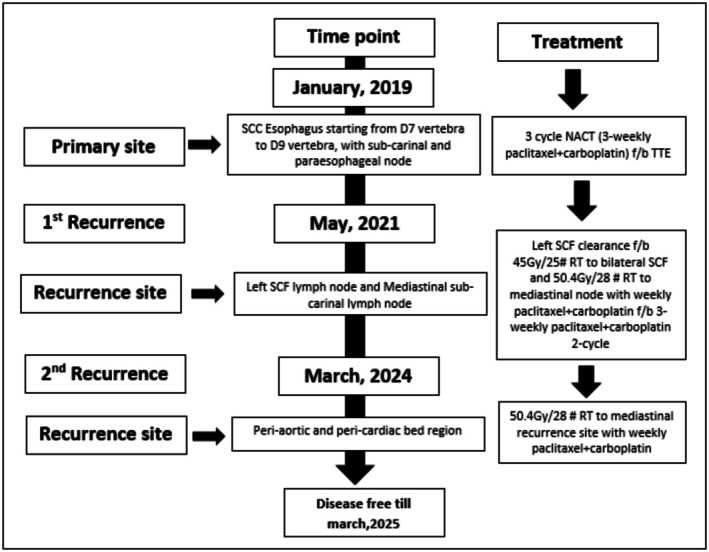
Summary of the case with time point of recurrences and treatment received.

At her most recent follow‐up in March 2025, the patient was reported to have no long‐term treatment‐related toxicity and continued to tolerate a solid diet. UGIE performed on January 22, 2025 was normal. This case is notable for its exceptional long‐term survival, despite two locoregional recurrences, both successfully managed with curative intent using multimodality treatment, including two rounds of concurrent chemoradiotherapy (Refer Figure 6). Importantly, the patient experienced minimal acute and no significant long‐term toxicity (Refer Table 2), retained good quality of life, and demonstrated durable disease control—an inspiring example of the potential benefits of aggressive but well‐tailored salvage.

## 5 Discussion

The treatment landscape of thoracic esophageal cancer has significantly evolved over the past decade. While radical surgery remains the cornerstone of management, locoregional and distant recurrences are still common in the majority of patients [7]. To improve long‐term disease control and reduce recurrence rates, multimodality treatment strategies incorporating chemotherapy, radiotherapy, or both have been employed in both neoadjuvant and adjuvant settings [3, 4, 5, 6].

However, there remains a lack of global consensus on the optimal neoadjuvant approach—whether neoadjuvant chemotherapy (NACT) or neoadjuvant chemoradiotherapy (nCRT) is superior. Practices vary significantly between Western countries and Asian nations such as Japan.

The landmark CROSS trial [3] established the benefit of nCRT before radical surgery in patients with locally advanced thoracic esophageal cancer, demonstrating improvements in R0 resection rates, pathological complete response (pCR), and overall survival (OS). Several subsequent trials [8, 9, 10] and a meta‐analysis [11] further reinforced the role of nCRT in this setting. In contrast, the JCOG 9907 trial from Japan showed that preoperative NACT significantly improved 5‐year OS compared to surgery alone [12].

However, there are no direct head‐to‐head comparisons between NACT and nCRT followed by surgery, making both approaches acceptable standards of care for middle thoracic esophageal squamous cell carcinoma (SCC). In this context, our patient was treated with three cycles of NACT followed by transthoracic esophagectomy (TTE) for middle thoracic esophageal SCC.

Despite definitive treatment, both locoregional and distant recurrences are common in esophageal cancer [13], often occurring within the first few years after therapy [14, 15]. Our patient also developed a regional recurrence within 2 years, consistent with this pattern.

There is no universally accepted guideline for managing regional nodal recurrence, and treatment strategies vary. Some centers advocate for metastectomy followed by adjuvant therapy if the lesion is surgically resectable [16], while others favor salvage chemoradiotherapy [17]. In our case, the patient underwent surgical nodal clearance of the left supraclavicular fossa (SCF) node, followed by definitive chemoradiotherapy targeting both the SCF and mediastinal nodal regions.

In some recent retrospective studies, it was shown that salvage radiotherapy or chemoradiotherapy is a viable option for loco‐regional recurrence after curative esophagectomy. Salvage radiotherapy has a better survival outcome without affecting quality of life [18, 19]. Some recent retrospective studies also showed promising results for survival outcome and safety of stereotactic radiation therapy (SRT) for oligometastatic locoregional or distant recurrences. Locoregional recurrence patients have longer survival than distant metastatic patients who underwent SRT [20].

Survival after a second regional recurrence of thoracic esophageal SCC is typically poor. Delivering curative‐intent re‐irradiation in such cases is extremely challenging due to the cumulative radiation dose limits of organs at risk (OARs), particularly when the recurrence occurs within or adjacent to previously irradiated fields.

The literature contains limited documentation of cases where patients with multiple regional recurrences underwent full‐dose re‐radiotherapy with minimal toxicity and long‐term survival. In our patient, we proceeded with curative‐intent re‐chemoradiotherapy for the second recurrence. During planning, the previous radiotherapy dose distribution was overlaid onto the new planning CT to avoid high‐dose overlap (Refer Figure 4a,b), and careful attention was given to cumulative OAR dose constraints to minimize toxicity (Refer Table 1).

**TABLE 1:** Below table shows OARs dose (EQD2) achieved during primary RT and re‐RT and cumulative dose and volumetric constraint.

OARs	EQD2 (of 1st RT)	EQD2 (of re‐RT)	EQD2 cumulative^a^
Spinal Cord *D*_max_	25.13 Gy	9.16 Gy	34.57 Gy
Lungs *D*_mean_	1.39 Gy	5.66 Gy	6.76 Gy
Lungs V20 Gy	1.82%	5.91%	—
Lungs V5 Gy	13.21%	66.80%	—
Heart *D*_mean_	2.26 Gy	8.29 Gy	11.75 Gy
Heart V20 Gy	7.14%	14.13%	—
Heart V30 Gy	4.05%	7.05%	—
TRACHEA/BRONCHUS *D*_mean_	31.97 Gy	14.91 Gy	55.75 Gy
TRACHEA *D*_max_	54.17 Gy	52.53 Gy	101.74 Gy
Gastric conduit *D*_mean_	4.49 Gy	9.7 Gy	14.32 Gy
Gastric conduit *D*_max_	46.73 Gy	53.7 Gy	95.48 Gy
Great vessels *D*_mean_	18.79 Gy	16.24 Gy	37.21 Gy
Great vessel *D*_max_	54.00 Gy	52.19 Gy	99.14 Gy

^a^ Both the 1st and re‐RT planning CT rigid registration was done, then deformable registration was done, then plan sum was generated with both the plans and evaluated for cumulative EQD2 (equivalent dose at 2 Gy per fraction).

The patient is currently disease‐free and on 1‐year follow‐up post‐second recurrence and after receiving two rounds of full‐dose curative‐intent radiotherapy in the mediastinal region, without developing any Grade ≥ 2 toxicities (Refer Table 2).

**TABLE 2:** Below table shows acute and late toxicity.

Toxicity	CTCAE v5.0 grade
*Acute*	
Dysphagia	Grade 1
Odynophagia	Grade 2
Fatigue	Grade 1
Skin reaction	Grade 1
Lung toxicity (productive cough, hemoptysis)	Grade 1
Cardiac toxicity (chest pain, arrhythmia)	Grade 1
*Late*	
Radiation‐induced pneumonia, hemoptysis	Grade 1
Radiation‐induced cardiomyopathy	Grade 1
Gastric conduit stricture	Grade 1
Aero‐digestive fistula	Grade 1
Fatigue	Grade 1

Abbreviation: CTCAE, common terminology criteria for adverse events.

Due to the lack of evidence of re‐irradiation in the mediastinal region we compare our survival and toxicity data with thoracic region re‐RT data. Available retrospective single institute data of re‐RT in the thoracic region show that re‐RT is safe and improve survival without increasing late toxicity. Local progression‐free survival was 12.9 months and overall survival was 31.4 months after re‐RT [21]. In our case also after 12 months post re‐RT, the patient is still disease‐free and alive.

In a resource‐constrained setting like India, nutritional challenges in gastrointestinal cancers are significant, and stricture formation in the neo‐esophagus following radiotherapy is a common cause of dysphagia and nutritional compromise [22]. In our case, no gastric conduit stricture was observed despite two courses of high‐dose thoracic radiotherapy, owing to the use of a highly conformal radiotherapy technique, which successfully spared the neo‐esophagus and surrounding critical structures.

Chemo‐radiotherapy is a very good option for the definitive treatment of regional nodal recurrence. Delivering the definitive dose of radiotherapy twice to the mediastinal region is very difficult due to the tolerance of OARs. Reirradiation in the mediastinal region can be performed safely with minimal toxicity and long‐term survival if there is an acceptable time gap between two radiotherapy periods, conservative tumor volumes, and optimization of radiation planning with a minimum dose to critical OARs (refer Table 1). Adjuvant chemotherapy can be used judiciously to consolidate response and minimize toxicity.

## Author Contributions

**Ajay Kumar Choubey:** resources, writing – original draft, writing – review and editing. **Pritam Mondal:** writing – original draft. **Shubham Dokania:** resources, writing – review and editing. **Sambit Swarup Nanda:** resources, writing – review and editing. **Shreya Jain:** resources, writing – review and editing. **Minesh Patel:** resources, writing – review and editing. **Ashutosh Mukherji:** resources, writing – review and editing. **Satyajit Pradhan:** resources, writing – review and editing.

## Funding

The authors have nothing to report.

## Ethics Statement

The authors have nothing to report.

## Consent

Written informed consent was obtained from the patient to publish this report in accordance with the journal's patient consent policy.

## Conflicts of Interest

The authors declare no conflicts of interest.

## Data Availability

The data that support the findings of this study are available on request from the corresponding author. The data are not publicly available due to privacy or ethical restrictions.

## References

[1] F. Bray , M. Laversanne , H. Sung , et al., “Global Cancer Statistics 2022: GLOBOCAN Estimates of Incidence and Mortality Worldwide for 36 Cancers in 185 Countries,” CA: A Cancer Journal for Clinicians 74 (2024): 229–263. 38572751 10.3322/caac.21834

[2] R. Siegel , E. Ward , O. Brawley , and A. Jemal , “Cancer Statistics, 2011: The Impact of Eliminating Socioeconomic and Racial Disparities on Premature Cancer Deaths,” CA: A Cancer Journal for Clinicians 61, no. 4 (2011): 212–236. 21685461 10.3322/caac.20121

[3] P. van Hagen , M. C. C. M. Hulshof , J. J. B. van Lanschot , et al., “Preoperative Chemoradiotherapy for Esophageal or Junctional Cancer,” New England Journal of Medicine 366, no. 22 (2012): 2074–2084. 22646630 10.1056/NEJMoa1112088

[4] J. Hoeppner , T. Brunner , F. Lordick , et al., “Prospective Randomized Multicenter Phase III Trial Comparing Perioperative Chemotherapy (FLOT Protocol) to Neoadjuvant Chemoradiation (CROSS Protocol) in Patients With Adenocarcinoma of the Esophagus (ESOPEC Trial),” Journal of Clinical Oncology 42 (2024): suppl.LBA1. 10.1186/s12885-016-2564-y PMC4952147 27435280

[5] S. E. Al‐Batran , N. Homann , C. Pauligk , et al., “Perioperative Chemotherapy With Fluorouracil Plus Leucovorin, Oxaliplatin, and Docetaxel Versus Fluorouracil or Capecitabine Plus Cisplatin and Epirubicin for Locally Advanced, Resectable Gastric or Gastro‐Oesophageal Junction Adenocarcinoma (FLOT4): A Randomised, Phase 2/3 Trial,” Lancet 393, no. 10184 (2019): 1948–1957. 30982686 10.1016/S0140-6736(18)32557-1

[6] V. Oppedijk , A. van der Gaast , J. J. B. van Lanschot , et al., “Patterns of Recurrence After Surgery Alone Versus Preoperative Chemoradiotherapy and Surgery in the CROSS Trials,” Journal of Clinical Oncology: Official Journal of the American Society of Clinical Oncology 32, no. 5 (2014): 385–391. 24419108 10.1200/JCO.2013.51.2186

[7] T. Boerner , R. A. Carr , M. Hsu , et al., “Incidence and Management of Esophageal Cancer Recurrence to Regional Lymph Nodes After Curative Esophagectomy,” International Journal of Cancer 152, no. 10 (2023): 2109–2122. 36573352 10.1002/ijc.34417 PMC10006335

[8] T. N. Walsh , N. Noonan , D. Hollywood , et al., “A Comparison of Multimodal Therapy and Surgery for Esophageal Adenocarcinoma,” New England Journal of Medicine 335, no. 7 (1996): 462–467. 8672151 10.1056/NEJM199608153350702

[9] J. Tepper , M. J. Krasna , D. Niedzwiecki , et al., “Phase III Trial of Trimodality Therapy With Cisplatin, Fluorouracil, Radiotherapy, and Surgery Compared With Surgery Alone for Esophageal Cancer: CALGB 9781,” Journal of Clinical Oncology: Official Journal of the American Society of Clinical Oncology 26, no. 7 (2008): 1086–1092. 18309943 10.1200/JCO.2007.12.9593 PMC5126644

[10] H. Yang , H. Liu , Y. Chen , et al., “Neoadjuvant Chemoradiotherapy Followed by Surgery Versus Surgery Alone for Locally Advanced Squamous Cell Carcinoma of the Esophagus (NEOCRTEC5010): A Phase III Multicenter, Randomized, Open‐Label Clinical Trial,” Journal of Clinical Oncology: Official Journal of the American Society of Clinical Oncology 36, no. 27 (2018): 2796–2803. 30089078 10.1200/JCO.2018.79.1483 PMC6145832

[11] T. Kumar , E. Pai , R. Singh , et al., “Neoadjuvant Strategies in Resectable Carcinoma Esophagus: A Meta‐Analysis of Randomized Trials,” World Journal of Surgical Oncology 18, no. 1 (2020): 59. 32199464 10.1186/s12957-020-01830-x PMC7085863

[12] N. Ando , H. Kato , H. Igaki , et al., “A Randomized Trial Comparing Postoperative Adjuvant Chemotherapy With Cisplatin and 5‐Fluorouracil Versus Preoperative Chemotherapy for Localized Advanced Squamous Cell Carcinoma of the Thoracic Esophagus (JCOG9907),” Annals of Surgical Oncology 19, no. 1 (2012): 68–74. 21879261 10.1245/s10434-011-2049-9

[13] F. Lou , C. S. Sima , P. S. Adusumilli , et al., “Esophageal Cancer Recurrence Patterns and Implications for Surveillance,” Journal of Thoracic Oncology 8, no. 12 (2013): 1558–1562. 24389438 10.1097/01.JTO.0000437420.38972.fb PMC4066875

[14] C. Mariette , J. M. Balon , G. Piessen , et al., “Pattern of Recurrence Following Complete Resection of Esophageal Carcinoma and Factors Predictive of Recurrent Disease,” Cancer 97, no. 7 (2003): 1616–1623. 12655517 10.1002/cncr.11228

[15] E. Abate , S. R. DeMeester , J. Zehetner , et al., “Recurrence After Esophagectomy for Adenocarcinoma: Defining Optimal Follow‐Up Intervals and Testing,” Journal of the American College of Surgeons 210, no. 4 (2010): 428–435. 20347734 10.1016/j.jamcollsurg.2010.01.006

[16] K. Hirose , H. Saeki , Y. Nakashima , et al., “Successful Multidisciplinary Treatment Including Repeated Metastasectomy for Recurrent Squamous Cell Esophageal Carcinoma: A Case Report,” Surgical Case Reports 5 (2019): 72. 31053962 10.1186/s40792-019-0634-5 PMC6499842

[17] K. Fakhrian , N. Gamisch , T. Schuster , R. Thamm , M. Molls , and H. Geinitz , “Salvage Radiotherapy in Patients With Recurrent Esophageal Carcinoma,” Strahlentherapie und Onkologie 188, no. 2 (2012): 136–142. 22218502 10.1007/s00066-011-0023-x

[18] W. K. Cho , J. M. Noh , D. Oh , et al., “Salvage Radiotherapy for Loco‐Regional Recurrence of Esophageal Cancer Following Surgery,” Cancer Research and Treatment 57, no. 1 (2025): 165–173. 39054622 10.4143/crt.2024.191 PMC11729326

[19] A. Katano , T. Kiritoshi , S. Sawayanagi , et al., “Salvage Chemoradiotherapy for Loco‐Regional Recurrence of Esophageal Squamous Cell Carcinoma After Esophagectomy,” Journal of Clinical Medicine 14, no. 5 (2025): 1540. 40095468 10.3390/jcm14051540 PMC11899801

[20] A. Katano , M. Minamitani , S. Ohira , S. Sawayanagi , and H. Yamashita , “Stereotactic Body Radiation Therapy for Oligometastatic Recurrent Esophageal Squamous Cell Carcinoma: A Retrospective Cohort Study From a Single Tertiary Center,” Cancer Reports 8, no. 6 (2025): e70248. 40524140 10.1002/cnr2.70248 PMC12170229

[21] K. Sumita , H. Harada , H. Asakura , et al., “Re‐Irradiation for Locoregionally Recurrent Tumors of the Thorax: A Single‐Institution, Retrospective Study,” Radiation Oncology 11 (2016): 104. 27485533 10.1186/s13014-016-0673-z PMC4971641

[22] J. W. Kim , T. H. Kim , J. H. Kim , et al., “Predictors of Post‐Treatment Stenosis in Cervical Esophageal Cancer Undergoing High‐Dose Radiotherapy,” World Journal of Gastroenterology 24, no. 7 (2018): 862–869. 29467556 10.3748/wjg.v24.i7.862 PMC5807944

